# Evaluation of the quality of antenatal care using electronic health record information in family medicine clinics of Mexico City

**DOI:** 10.1186/1471-2393-14-168

**Published:** 2014-05-16

**Authors:** Svetlana V Doubova, Ricardo Pérez-Cuevas, Eduardo Ortiz-Panozo, Bernardo Hernández-Prado

**Affiliations:** 1 Epidemiology and Health Services Research Unit CMN Siglo XXI, Mexican Institute of Social Security, Mexico City, México; 2 Division of Social Protection and Health, Inter American Development Bank, Mexico City, México; 3 Center for Population Health Research, National Institute of Public Health, Cuernavaca, Mexico; 4 Institute for Health Metrics and Evaluation, University of Washington, Seattle, USA

**Keywords:** Antenatal care, Quality evaluation, Electronic health record, Family medicine clinics, Mexico

## Abstract

**Background:**

Evaluation of the quality of antenatal care (ANC) using indicators should be part of the efforts to improve primary care services in developing countries. The growing use of the electronic health record (EHR) has the potential of making the evaluation more efficient. The objectives of this study were: (a) to develop quality indicators for ANC and (b) to evaluate the quality of ANC using EHR information in family medicine clinics (FMCs) of Mexico City.

**Methods:**

We used a mixed methods approach including: (a) in-depth interviews with health professionals; (b) development of indicators following the RAND-UCLA method; (c) a retrospective cohort study of quality of care provided to 5342 women aged 12–49 years who had completed their pregnancy in 2009 and attended to at least one ANC visit with their family doctor. The study took place in four FMCs located in Mexico City. The source of information was the EHR. SAS statistical package served for programing and performing the descriptive statistical analysis.

**Results:**

14 ANC quality indicators were developed. The evaluation showed that 40.6% of women began ANC in the first trimester; 63.5% with low-risk pregnancy attended four or more ANC visits; 4.4% were referred for routine obstetric ultrasound, and 41.1% with vaginal infection were prescribed metronidazole. On average, the percentage of recommended care that women received was 32.7%.

**Conclusions:**

It is feasible to develop quality indicators suitable for evaluating the quality of ANC using routine EHR data. The study identified the ANC areas that require improvement; which can guide future strategies aimed at improving ANC quality.

## Background

Evidence-based antenatal care (ANC) is effective in reducing the unfavorable health outcomes during pregnancy and postpartum [1,2]. With this aim, the World Health Organization (WHO) recommends the introduction of standards of care and improvements in the ANC process [3]. Evaluation of quality of care is a key component of the process, allowing the identification of the areas of opportunity and potential gains of introducing improvements. In low-resource settings, assessment of the quality of care is not conducted in a routine basis because it requires defined indicators and access to relevant clinical information.

In Mexico, ANC is institutionalized in public healthcare systems; it is among the five top causes of ambulatory care, reaching ~98% coverage [4]. However, the rates of maternal and neonatal complications and deaths during pregnancy and delivery are higher than expected [5,6]. The Mexican Institute of Social Security (IMSS; after its name in Spanish) provides health care to nearly 47 million people (40% of Mexico’s population). IMSS provides ANC in its family medicine clinics (FMCs) and obstetric care in its hospitals. This institution has developed clinical guidelines and standardized processes of ANC; it also uses the electronic health record (EHR) for the routine provision of healthcare. Nevertheless, IMSS lacks validated indicators to evaluate comprehensively the quality of ANC and has not used EHR information to systematize this process [7].

Under these circumstances, this study has two objectives: first to develop quality indicators for ANC, and second to evaluate the quality of ANC using EHR information in family medicine clinics (FMCs) of Mexico City.

## Methods

The study took place in four IMSS FMCs in Mexico City, selected by convenience. The clinics had similar infrastructure in terms of examining rooms, laboratory, pharmacy, EHR and institutional clinical guidelines. The clinics refer patients to secondary level hospitals as needed through the IMSS referral system. The population covered by these clinics was ~585,500 people. The staff was comprised of family doctors, nurses, social workers and dietitians. The FMCs provide ambulatory care in the morning and evening shifts in its 103 family doctors’ offices (15–30 per clinic). All clinics above 10 examining rooms at IMSS are fairly similar.

A two-stage mixed methods approach was used; in particular, we applied the instrument development model of an exploratory sequential design [8]. The first stage of this design gathered qualitative information through interviews with key informants in the health facilities. This information guided the quantitative phase which consisted in the development of the items (indicators of quality of care) for the second stage of the study.

### Stage 1: Development of the list of quality of care indicators for ANC

This stage took place from November 2009 to February 2010. The list of quality indicators was built following two consecutive steps: (i) conducting in-depth interviews with FMC health professionals and (ii) using a modified version of the RAND/UCLA Appropriateness Method [9].

In-depth interviews attempted to capture the sequence and content of the ANC process and perceived barriers in the use of the EHR at IMSS facilities. The key informants were three heads of clinical department, three family doctors, three nurses and three social workers with ≥10 years of experience providing ANC. Respondents were selected according to their experience and previous participation in implementing ANC-related programs. The researchers conducted in-depth interviews based on eight pre-defined topic questions. All interviews were conducted in-person at the FMCs.

The RAND/UCLA Appropriateness Method is a suitable tool for developing indicators; it combines a literature review of scientific evidence and expert group validation [9]. The literature review collected information about the care that pregnant women should receive. Available electronic repositories of clinical guidelines were searched, which included international [10-12] and IMSS’ guidelines [13], as well as the indicators that the RAND group [14] have proposed for ANC. The keywords for the search were “antenatal care”, “prenatal care”, “guidelines”, “quality indicators”, “family medicine” and “general medical practice”.

A preliminary list of indicators was developed with the participation of two family doctors, two obstetrician-gynecologists and two health systems researchers, a group of experts with experience in constructing indicators. The group received information about the objectives of the study, a preliminary list of indicators, and the previously mentioned guidelines. All participants rated the validity and feasibility of the preliminary indicators by using Shekell’s criteria [15]. As it has been the use in other studies, o those indicators with a score >7 were taken into account [15]. After three rounds of discussion in the group, a final list of indicators was produced, which covered five areas: (i) initiation and number of antenatal visits; (ii) health education; (iii) screening for pre-existing diseases and complications; (iv) supplementation; and (v) treatment and referral for specialized care.

### Stage 2: Evaluation of the quality of care

A retrospective cohort study was conducted with women aged 12–49 years who had completed their pregnancy in 2009 and attended to at least one ANC visit with their family doctor in 2008–2009, as indicated above. The source of information was the EHR. The women should have registered the diagnosis of pregnancy according to the codes Z321, Z34, Z35, Z36 that are in the 10th revision of the International Classification of Diseases.

The study variables were:

A) Women’s general characteristics: age, schooling, marital status, employment and IMSS insurance status (insured or beneficiary).

B) Medical and reproductive history: pregnancy risk, pre-existing comorbidity, number of pregnancies, history of premature births and low-birth weight, Rh-negative multigravid with Rh-positive partner.

C) Nutritional status: The pre-pregnancy nutritional status of women was classified as normal weight (body mass index **(**BMI**)** 18.5-24.9 kg/m^2^), overweight (BMI 25–29.9 kg/m^2^) and obese (BMI ≥30.0 kg/m^2^), pre- and post-pregnancy weights, gestational weight gain and gains above the range that the Institute of Medicine recommends: BMI <18.5 kg/m^2^, a gain of 12.5-18 kg, BMI 18.5-24.9 kg/m^2^ a gain of 11.5-16 kg, BMI 25–29.9 kg/m^2^ a gain of 7.0-11.5 kg, BMI ≥ 30 kg/m^2^ a gain of 5–9 kg [16].

D) Pregnancy complications: urinary tract infections, preeclampsia, gestational diabetes, hemorrhage, fetal death and bacterial vaginosis or trichomoniasis.

E) Estimation of the quality of ANC involved the indicators constructed in the first stage. To obtain a comprehensive evaluation of the ANC process, we also estimated the percentage of care that the women received in relation to the recommended ANC [17]. The recommended healthcare was estimated as a proportion in which the numerator was the sum of all the indicators that a woman received and the denominator was the total number of indicators for which the woman was eligible.

### Data programming and statistical analysis

We used structured query language (SQL) to retrieve data from EHR tables and create a new analytic database. Non-plausible values were identified and dropped from the analysis, for example, height <100 cm and weight <18 kg, systolic blood pressure <50 mmHg and diastolic blood pressure <40 mmHg. The maximum values were predefined in the EHR.

SAS statistical package (version 9.2) was used to program the set of complex variables for the quality of ANC indicators and obtain the descriptive statistics of the variables. The units of analysis were pregnant women. There were three variables with missing data (marital status, occupation and pre-pregnancy nutritional status) that were reported. The other variables did not have any missing data.

### Ethical issues

This article forms part of the study that was aimed at developing a system for individualized evaluation and improvement of healthcare quality of the eight most frequent causes of visits at IMSS FMCs through using electronic health record information. The study protocol was approved by the IMSS National Research and Ethics Committees (CNIC: 2008-785-008). The data for the analysis was obtained in each FMC. The study only used the EHR information. All identification data of the women in the EHR were de-identified; thus, it was not possible to trace any of the data to the actual individual. For this reason the informed consent was not requested.

## Results

Stage1. In-depth interviews about the sequence of ANC process at IMSS indicated that ANC begins in FMCs, where family doctors determine whether the woman has low or high risk pregnancy. Women with a low-risk pregnancy receive ANC at the FMCs from the healthcare team, which includes the family doctor, a maternity nurse, a nutritionist and a social worker. The health care team should provide four group sessions of health education to pregnant women. The topics covered include progression of pregnancy, pregnancy complication warning signs, parenthood and breathing techniques for labor and delivery, as well as post-partum information. The high-risk pregnant women, or those who develop severe complications during pregnancy such as preeclampsia, are referred for specialized care. All pregnant women deliver at IMSS hospitals.

The EHR has functional and organizational limitations. Only the family doctors enter routinely the data in the EHR. The other members of the health team do not register or register sporadically the clinical information due to the lack of clear organizational indications; thus, they register their activities (immunization, educational activities, etc.) in monthly record sheets and in an ad-hoc preventive care electronic system not linked to the EHR. Furthermore, the family doctors face technical barriers to register the information, such as system interruptions and flaws in the design of the EHR, which impairs swift navigation from one application to another; also the system does not have locks or warnings against implausible data. The EHR lacks a particular application for registering information on delivery and post-partum, including the condition of newborns.

The findings from the in-depth interviews and literature review resulted in a preliminary list of 27 indicators, from which the group of experts discarded thirteen indicators because these were not feasible or informative for IMSS (Table 1, part B). The indicator *urine protein test for early pre-eclampsia detection* was discarded because it is not routinely used at FMCs. There is evidence supporting the benefit to screen all pregnant women for virological measles, HIV and hepatitis B; nonetheless, such tests are not available at IMSS FMCs. To mitigate this limitation, the IMSS clinical guideline recommends identifying those patients at risk for these diseases in primary care and referring them to the second or third level of care. Consequently, these indicators were discarded. Pregnancy risk assessment, measurement of blood pressure, weight and symphysis-fundal height were also discarded because of the lack of variability in these indicators, since such procedures must be entered into the EHR before proceeding with other fields of information. The remaining five indicators were discarded because they were not registered routinely at the EHR. The validation process concluded with 14 indicators that were possible to construct using the EHR data. The indicator “pregnant women who were referred to fasting plasma glucose test during the first two ANC visits and between weeks 24 and 28 of gestation” was considered as a surrogate of the international recommendation of performing oral glucose tolerance test, which IMSS guidelines currently do not recommend for pregnant women.

**Table 1:** List of quality of care indicators

	**Part A. accepted indicators**	
	**Initiation and number of antenatal visits**	
**1**	Percentage of pregnant women who began ANC during the first trimester of gestation	
**2**	Percentage of women with low risk pregnancy^†^ who at the end of the pregnancy had at least four ANC visits	
	**Health education**	
**3**	Percentage of pregnant women who had documented educational activities provided by the maternity nurse or social worker	
**4**	Percentage of overweight/obese (pre-pregnancy BMI ≥ 25 kg/m2) pregnant women who had documented nutritional counselling provided by the nutrition service	
	**Screening**	
**5**	Percentage of pregnant women who were referred to or had documented Rh and blood group test	
**6**	Percentage of pregnant women who were referred to haemoglobin test during the first two ANC visits	
**7**	Percentage of pregnant women who were referred to fasting plasma glucose test during the first two ANC visits and between weeks 24 and 28 of gestation	
**8**	Percentage of pregnant women who were referred to obstetric ultrasound between weeks 18 and 22 of gestation	
**9**	Percentage of pregnant women who were referred to VDRL test (syphilis screening) during the first two ANC visits	
	**Nutritional supplementation**	
**10**	Percentage of pregnant women who had prescription of folic acid during the first trimester of gestation	
	**Treatment and referrals to the obstetrician-gynecologist**	
**11**	Percentage of pregnant women diagnosed with bacterial vaginosis or trichomoniasis, who had vaginal metronidazol prescription in adequate doses and duration	
**12**	Percentage of pregnant women with systolic blood pressure ≥ 140 mmHg, or diastolic blood pressure ≥ 90 mmHg who was referred to the second or third level of care	
**13**	Percentage of pregnant women with pre-existing degenerative chronic disease (diabetes, hypertension, lupus, heart disease) who were referred to the second or third level of care	
**14**	Percentage of pregnant women between 20–32 weeks with symphysis-fundal height 4 cm less than indicated by their gestational age, who were referred to ultrasound or another level of care	
	**Part B. Discarded indicators**	**Reasons of elimination**
1. Percentage of pregnant women who had documented risk assessment at the first ANC visit.		These processes of care correspond to the mandatory fields in the EHR, so the family doctor and other health care professional cannot move along EHR without fill out these fields.
2. Percentage of pregnant women who had their blood pressure measured at each ANC visit.		
3. Percentage of pregnant women who had their weight measured at each ANC visit.		
4. Percentage of pregnant women who had their symphysis-fundal height measured at each ANC visit from 24 weeks of gestation.		
5. Percentage of pregnant women who underwent a urine protein test at each visit from 20 weeks of gestation.		The urine protein test is not routinely performed in most IMSS family medicine clinics.
6. Percentage of pregnant women who underwent screening for HIV.		Such tests are not available at IMSS FMCs. To mitigate this limitation, the IMSS clinical guideline recommends identifying those patients at risk for these diseases in primary care and referring them to the second or third level of care.
7. Percentage of pregnant women who underwent screening for hepatitis B.		
8. Percentage of pregnant women who underwent screening for measles.		
9. Percentage of smoking pregnant women who underwent smoking cessation counselling.		The information is not routinely registered at the EHR.
10. Percentage of pregnant without previous tetanus toxoid immunization women who had documented indication for this immunization during the first trimester of gestation.		
11. Percentage of multigravid Rh negative pregnant women with		
Rh-positive partner who was referred to the second or third level of care before week 28 of gestation.		
12. Percentage of pregnant women who had premature birth (≤37 weeks) newborn.		
13. Percentage of pregnant women who had low birth weight (<2500 g) newborn.		

^†^Low risk pregnancy: <4 points at the obstetric risk scored system of EHR.

Stage 2 In 2009, 5540 women completed a pregnancy, in which 5342 (96.1%) had at least one ANC visit and the rest (3.9%) had the diagnosis of pregnancy without evidence of attendance at ANC in the four clinics.

Table 2 gives the general characteristics of the participating women. Out of 5342 women, 91% were between 20 and 39 years, with a mean age of 28.8 years at the time of the first ANC visit; 72% of them had completed high school and college. Data on marital status were missing in 87.5% of cases; 64.8% were insured, which means that they had a formal job. Nevertheless, information about their type of occupation was missing (73%), but the recorded data shows that most women worked as administrative staff (9.5%).

**Table 2:** General characteristics of the women in the study

**Variables**	**n = 5342%**
**Age group**	
<20 years	5.9
20–29 years	49.7
30–39 years	41.3
40–49 years	3.1
**Schooling**	
Primary school or less	4.9
Secondary school	22.7
High school	38.9
University degree	33.5
**Marital status**	
Married or partnership	9.3
Single or divorced	3.2
Missing data	87.5
**Insurance status**	
Insured	64.8
Beneficiary	35.2
**Occupation**	
Professionals	7.6
Administrative staff	9.5
Service workers and sailors	3.1
Arts and craft artisans	0.7
Elementary occupations	2.4
Housewife	3.0
Student	0.7
Missing data	73.0

Table 3 shows the medical and reproductive history: Less than 1% had diabetes or hypertension prior to pregnancy; and 71.6% were nulliparous. Among multigravid women, the median of gestations was 1 (range 1–7). Only a small percentage (2.3%) had documented a previous premature birth, or low birth-weight children (1.9%). There was no record of Rh-negative multigravid with an Rh-positive partner. According to the family doctors, evaluation at the first ANC visit showed that 6.5% had a high-risk pregnancy.

**Table 3:** Medical, reproductive history, nutritional status and pregnancy complications

**Variables**	**n = 5342%**
**Pre-existing comorbidity**	
Diabetes	0.4
Hypertension	0.7
**Gravidity**	
Nulliparous	71.6
**Reproductive history of multigravid women**	**n = 1517**
Number of gestations median (minimum-maximum)	1 (1–7)
Prior premature birth (<37 weeks)	2.3
Prior low birth weight (<2500 g)	1.9
Rh-negative multigravid	0.8
Rh-negative multigravid with Rh-positive partner	0.0
High risk pregnancy*	6.5
**Nutritional status and gestational weight gain**^† ^	
**Pre-pregnancy nutritional status**	**n = 3400**
Under weight	1.9
Normal weight	46.5
Overweight	34.8
Obesity	16.8
Pre-pregnancy weight, kg, mean (standard deviation)	63.6 (12.1)
Post-pregnancy weight, kg, mean (standard deviation)	**n = 1714**
	71.1 (11.9)
Gestational weight gain, kg, mean (standard deviation)	8.4 (5.4)
High gestational weight gain, %	15.4
**Pregnancy complications in current pregnancy**	**n = 5342**
Preeclampsia	5.0
Gestational diabetes	0.1
Hemorrhage	0.6
Fetal deaths	0.6
Bacterial vaginosis, trichomoniasis	1.8

*Pregnancy risk, registered by family doctor at the first ANC visit; ^†^Only 63% of women had registered pre-pregnancy weight and 32.1% post-pregnancy weight.

Pre- and post-pregnancy weights were poorly registered. Only 63% of women had registered a pre-pregnancy weight and 32.1% a post-pregnancy weight. This lack of information about weight was due to women not attending healthcare before being pregnant, and there were other cases in which this information was not registered at the end of pregnancy. Furthermore, 3% had non-plausible values for this variable. Among women whose weight was registered, almost 50% were overweight (overweight: 34.8%, obesity: 16.8%). The average gestational weight gain was 8.4 kg, with 15.4% having a high weight gain. The complications of pregnancy registered in the EHR were pre-eclampsia (5%) and bacterial vaginosis (1.8%); less than 1% had bleeding and fetal death.

Table 4 shows the evaluation of the quality of ANC in the five areas of ANC. In the area that addresses the initiation and number of antenatal visits, 41% of women began ANC in the first trimester, 42% in the second and 17% in the third trimester (not shown in the table); 63% of women with low risk pregnancy had made four or more ANC visits. In the area of health education, 21% were referred for educational activities with a maternity nurse or social worker, and only 26% of women who were overweight or obese from the onset of pregnancy received nutritional counseling. The area of screening for pre-existing diseases and complications showed that the most common screening tests were for anemia (42%), gestational diabetes (41%) and syphilis (40%), whereas only 4.4% were referred for obstetric ultrasound. Regarding the area of supplementation, 64% had received folic acid supplementation during the first trimester. The area of treatment for complications and referral for specialized care showed that 92% of women with a pre-existing chronic condition, and 69% of women diagnosed with hypertension during pregnancy, were referred for specialized care, yet only 45% of those with mild conditions (such as vaginal infection) received treatment. On average, women received 32.7% of the recommended ANC.

**Table 4:** Quality of care

**Indicators**	**%**
**Initiation and number of antenatal visits**	
Pregnant women who began ANC during the first trimester of gestation	**n = 5342**
	40.6
Women with low risk pregnancy† who at the end of the pregnancy had at least 4 ANC visits	**n = 4300**
	63.5
**Education for health**	
Pregnant women with documented educational activities provided by the maternity nurse or social worker	**n = 5342**
	20.7
Overweight/obese (pre-pregnancy BMI ≥ 25 kg/m2) pregnant women who had documented nutritional counseling provided by the nutrition service	**n = 1754**
	26.0
**Screening**	**n = 5342**
Pregnant women referred to or had documented Rh and blood group test	23.9
Pregnant women referred to hemoglobin test during the first 2 ANC visits	41.9
Pregnant women referred to fasting plasma glucose test during the first 2 ANC visits and between weeks 24 and 28 of gestation	41.1
Pregnant women referred to obstetric ultrasound between weeks 18 and 22 of gestation	4.4
Pregnant women referred to VDRL test (syphilis screening) during the first 2 ANC visits	40.1
**Supplementation**	**n = 2169**
Pregnant women prescribed folic acid during the first trimester of gestation	64.6
**Treatment and references to the obstetrician-gynecologists consultation at the second level of care**	
Pregnant women diagnosed with bacterial vaginosis or trichomoniasis who had vaginal metronidazol prescription	**n = 95**
	45.3
Pregnant women with systolic blood pressure ≥140 mmHg, or diastolic ≥ 90 mmHg referred to the second or third level of care	**n = 291**
	69.1
Pregnant women with pre-existing degenerative chronic disease (diabetes, hypertension, lupus, heart disease) referred to the second or third level of care	**n = 54**
	92.6
Pregnant women between 20–32 weeks with symphysis-fundal height 4 cm less than indicated by their gestational age, referred to ultrasound or another level of care	**n = 1445**
	60.3
**Percentage of recommended care**^**††**^, mean (standard deviation)	**32.7 (22.2)**

n: indicates the number of women eligible for the evaluated indicator; †Low risk pregnancy: <4 points at the obstetric risk scored system of EHR; ^††^Proportion of antenatal care procedures that a woman received relative the total number of indicators for which she was eligible.

## Discussion

The study shows the feasibility of developing indicators to assess the quality of ANC using existing data from the EHR. It also identifies the quality of ANC as having a wide margin for improvement in all areas of evaluation comprising the set of indicators, thus signaling that there are unmet needs in the care of pregnant women. Furthermore, the findings show that the process of ANC should be renovated at IMSS FMCs. Some of the indicators that were discarded signal scarcity of resources and obsolescence in criteria to provide evidence-based ANC.

The method of developing the indicators is robust. The modified version of the RAND/UCLA Appropriateness Method combines scientific evidence and expert consensus, allowing the construction of scientifically rigorous and contextually suitable indicators that permit valid judgments and reproducible evaluations [9].

The use of ANC indicators has been previously reported in the literature; we identified one study that reported the method followed to design and validate the indicators [17]. Other studies only reported the authors’ selection of the indicators from a list of ANC procedures, without reporting the validation method [18-21]. For example, a study in Mexico proposed a list of 12 ANC procedures that comprised history-taking and diagnosis (blood and urine tests, and history of bleeding and discharge), physical examination (blood pressure, weight, and uterine height), and preventive procedures (tetanus toxoid immunization, iron supplements, family planning and breastfeeding counselling, and use of the health card) [18]. In Vietnam the investigators defined a typical minimum ANC content: seven items on bio-medical assessments (body weight, blood pressure, fundal height, fetal heart rate, vaginal examination, urine testing, and ultrasound), four items on care provision (tetanus toxoid immunization, provision of tablets or advice on iron/folate supplement, malaria prevention, and preparation for safe delivery), and two items on health promotion/education (resting and nutrition) [19]. These indicators addressed a variable number of the ANC processes, and were context-specific.

The quality indicators cannot simply be transferred from one country to another or from one healthcare system to another. Before using them, it is advisable to learn about their applicability and carry out a rigorous validation [22]. In our study the indicators of RAND Group were the referent to develop the IMSS indicators, which in turn were assessed for their validity and feasibility. Comparing with RAND indicators (39 indicators) the list of IMSS indicators was shorter, as several indicators were considered not applicable, given that the institution did not perform some antenatal care procedures due to resource constraints, such as screening for HIV and hepatitis B, or the protein-urine test.

Epidemiological information suggests that, in Mexico, little has been achieved to improve maternal and newborn health, specifically ANC [23]. The lack of systematic evaluation of ANC quality impairs proper identification of areas needing improvement. Although this study was conducted in a reduced number of facilities, the set of indicators that were developed and validated could be seen as a preliminary effort to systematize ANC evaluation, and guide the implementation and monitoring of large-scale programs aimed at improving the quality of ANC care.

Moreover, the availability of the EHR increases the feasibility of scaling up the scope and rigorousness of these evaluations. Nevertheless, the published experience of evaluating quality of care using EHR data is limited in the Latin- American region [24,25]. The results of this study stress that the overall quality of ANC was poorly achieved, given that pregnant women received only a third part of the recommended care. This percentage was below than the reported in the United States [17] where it reached 73% and in another study conducted in Mexico, where >80% of pregnant women received the recommended care [18]. In contrast, pregnant women in Vietnam [19] and Brazil [20] attained 1.8% and 15% of the recommended care, respectively; yet comparison is difficult because of differences in the analyzed indicators and the source of information, given that these studies did not analyze EHR data.

Flaws were observed in all areas of provision of ANC, and this study suggests specific areas that could be improved. Early initiation of ANC increases the likelihood of timely identification and appropriate management of pregnancy complications. In our study, less than 50% of women began ANC during the first trimester of pregnancy. This result is much lower than reported in high-income countries [26] where 66-90% begins ANC in the first trimester. Our results are similar to those from Brazil and Vietnam [19,20]. This indicates the need to train health professionals and inform reproductive age women about the importance of early ANC.

In low-risk pregnancies, four ANC visits are recommended for women to receive required screening, immunization and educational activities [10]. In the present study 36% of low-risk pregnant women had made less than four ANC visits. In fact, it is not just the number of visits, but their content that matters in providing high quality ANC. For example, according to clinical guidelines, most of the screening activities should be performed during the first ANC visit.

The key areas of health education considered to be effective in improving pregnancy outcomes include education about a healthy diet, prevention of accidents and early identification of warning signs of complications [10-13]. However, we found that only 20.7% had documented educational activities with a maternity nurse or a social worker, and 26% received nutritional counseling, despite that half of the pregnant women were overweight or obese from the onset of pregnancy. This finding indicates an unmet need for health education, nutritional care, and the issue of incomplete information in the EHR that should be seriously considered as part of any quality of care improvement program.

Screening for pre-existing diseases and complications requires further action that can be beneficial for pregnant women. Antenatal ultrasound for fetal abnormalities was used in only 4.4% of women; this study allows early detection of neural tube defects (NTDs) and intrauterine growth retardation, among other abnormalities, as well as being cost-effective [11]. Mexico has a high prevalence of NTDs [27], which justifies the need for ultrasound studies of pregnant women.

A low blood folate level is associated with increased risk of NTD, spontaneous abortion and low birth-weight [28]. Folic acid supplementation can reduce the incidence of NTDs by up to 70% [29]. Nevertheless, only 64% of women were prescribed folic acid, pointing out the need to reinforce supplementation of folic acid by educating health professionals and women of reproductive age.

There is a lack of continuity between the primary care setting and the specialized care for women who develop severe complications. In our study, only 69% of women with a blood pressure higher than 140/90 mmHg, and 60% of those with symphysis-fundal height 4 cm below the corresponding to their gestational age were referred for specialized care. The rates of gestational hypertension and low birth-weight are high among IMSS affiliates. For example, a study conducted in 2005 in Mexico City reported that 31% of maternal deaths were attributable to pre-eclampsia and 45% had received poor healthcare [30].

The indicator of women treated for bacterial vaginosis was only 45.3%. There is evidence of an association between bacterial vaginosis and an increased incidence of adverse pregnancy outcomes, such as premature rupture of membranes, preterm delivery and low birth weight [31]. Although a recent Cochrane review [32] provides little evidence that screening and treating all pregnant women with bacterial vaginosis will prevent preterm birth and its consequences. However the same review commented that, when screening criteria were broadened to include women with abnormal flora, there was a 47% reduction in preterm birth.

The study has several strengths. (i) The method of developing the indicators was robust and replicable. (ii) The EHR contains routinely collected data that made the evaluation feasible, and this was based on clinical information from the day-to-day provision of healthcare services; specifically for this study, the extraction and programming of the information allowed retrospective analysis of the data from 5342 pregnant women seen in a calendar year. (iii) The procedure is efficient and replicable and reduces errors in data collection making it convenient for complex healthcare systems, such as IMSS.

However, several limitations were noted. First, our measure of quality of ANC relies on the EHR data and therefore could overestimate or underestimate the quality of ANC. It is possible that some procedures might have been performed (e.g. referral to the dietitian) during the ANC visits without being registered or vice versa. For ethical and practical reasons, it would be impossible to use other strategies to document such procedures, e.g. direct observation or hidden patients. However, the procedures followed at IMSS and the experience of other studies conducted at IMSS suggests that health records from FMCs, like those included in this study, can accurately reflect the actions taken during ANC. Second, the scope of the evaluation of the quality of ANC is limited due to the lack of information regarding the delivery and post-partum period, including the conditions of the newborns. Finally, the study was conducted in four FMCs, which means that the results may not be representative of all the 1400 IMSS FMCs in Mexico. However, the methodology can be used to evaluate and to compare the quality of ANC in other health systems in Mexico or other countries that use EHRs for ANC.

## Conclusions

This study suggests that it is feasible to develop quality indicators and evaluate the quality of ANC using existing EHR data. The evaluation shows flaws in the provision of ANC, which can guide in a precise way the development of strategies aimed at improving the quality of care and the use of the EHR. The study provides a valuable experience for developing countries regarding the use of the EHR for quality of ANC evaluation.

## Competing interests

The authors declare that they have no competing interests.

## Authors’ contributions

SVD conducted the literature review, coordinated the development, definitions, and programming of the indicators, interpreted the data and wrote the article. RPC conceptualized the study, participated in the development of the indicators and contributed to drafting the article. EOP y BHP critically reviewed the manuscript for significant intellectual content. All authors approved the final manuscript.

## Pre-publication history

The pre-publication history for this paper can be accessed here:

http://www.biomedcentral.com/1471-2393/14/168/prepub
